# Near-Infrared Light-Triggered Thermo-responsive Poly(*N*-Isopropylacrylamide)-Pyrrole Nanocomposites for Chemo-photothermal Cancer Therapy

**DOI:** 10.1186/s11671-020-03444-4

**Published:** 2020-11-12

**Authors:** Ha Hee Shin, Hyung Woo Choi, Jae Hyun Lim, Ji Woon Kim, Bong Geun Chung

**Affiliations:** 1 Department of Biomedical Engineering, Sogang University, Seoul, Korea; 2 Department of Mechanical Engineering, Sogang University, Seoul, Korea

**Keywords:** Poly(N-isopropylacrylamide), Lower critical solution temperature, Pyrrole, Cancer targeting, Chemo-photothermal therapy

## Abstract

The combination therapy based on multifunctional nanocomposites has been considered as a promising approach to improve cancer therapeutic efficacy. Herein, we report targeted multi-functional poly(*N*-isopropylacrylamide) (PNIPAM)-based nanocomposites for synergistic chemo-photothermal therapy toward breast cancer cells. To increase the transition temperature, acrylic acid (AAc) was added in synthetic process of PNIPAM, showing that the intrinsic lower critical solution temperature was changed to 42 °C . To generate the photothermal effect under near-infrared (NIR) laser irradiation (808 nm), polypyrrole (ppy) nanoparticles were uniformly decorated in PNIPAM-AAc. Folic acid (FA), as a cancer targeting ligand, was successfully conjugated on the surplus carboxyl groups in PNIPAM network. The drug release of PNIPAM-ppy-FA nanocomposites was efficiently triggered in response to the temperature change by NIR laser irradiation. We also confirmed that PNIPAM-ppy-FA was internalized to MDA-MB-231 breast cancer cells by folate-receptor-mediated endocytosis and significantly enhanced cancer therapeutic efficacy with combination treatment of chemo-photothermal effects. Therefore, our work encourages further exploration of multi-functional nanocarrier agents for synergistic therapeutic approaches to different types of cancer cells.

## Introduction

Drug delivery system (DDS) is one of the powerful methods of administering a pharmaceutical compound to achieve a therapeutic effect in cancer therapy [1]. Although the goal of DDS is to deliver the active drug and accumulate in the desired area, the conventional DDS is often accompanied by severe side effect and inefficient therapeutic efficiency [2, 3]. To overcome these obstacles, a variety of nanocarriers, which have the ability to respond to specific internal or external stimuli including temperature, light, pH, electric field, oxidation–reduction, enzyme activity, and antigen concentration, have been developed for advanced DDS and used to induce sustained and controlled drug release [4–6].

One of the various stimuli, the thermo-responsive nanocarrier is a powerful approach to improve cancer treatment, because drugs into nanoparticles could be released at a certain temperature [7]. Besides, the advantage of thermo-sensitive nanoparticles could be combined with other stimuli to induce the complete cancer eradication [8, 9]. As a thermo-responsive nanoparticle, poly(*N*-isopropylacrylamide) (PNIPAM) has received the most attention, because it exhibits a phase transition at a lower critical solution temperature (LCST) of around 32 °C [10, 11]. Below the LCST, whole polymer network in PNIPAM exists in a swollen state due to the hydrogen bonds. On the other hands, PNIPAM transits to the hydrophobic state and hydrogen bonds are reduced, resulting in a collapse of the polymer network above the LCST [12, 13]. Compared to other nanoparticles, PNIPAM-based nanocarrier has the advantage of high drug encapsulation efficiency, controlled drug release capacity, and good biocompatibility [14]. However, due to the spontaneous drug release at body temperature, PNIPAM-based nanocarrier is not sufficient to use in advanced DDS. To surmount this, the previous studies added the organic acids (e.g., vinyl acetic, acrylic, and allyl acetic acid) in synthetic process of PNIPAM, resulting in the reduced side effects by the continuous drug release [15].

**Scheme 1.**
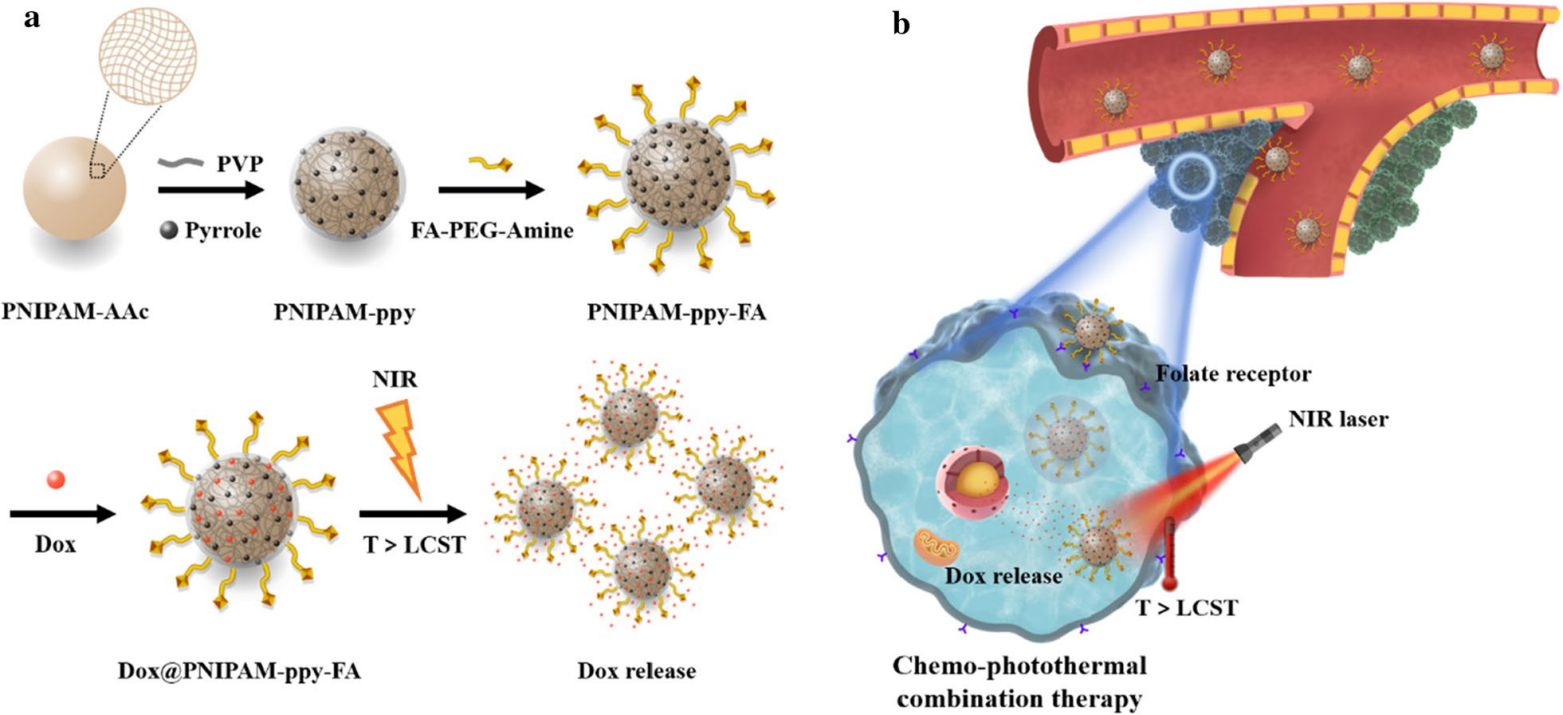
Schematic illustration of **a** synthesis of NIR light and thermos-triggered Dox@PNIPAM-ppy-FA nanocomposites and **b** application for enhanced chemo-photothermal combination therapy in breast cancer cells

Recently, a number of studies have focused on new therapeutic approaches that the integration of two or more stimuli triggered nanoparticles for enhanced cancer therapeutic efficacy [16–18]. For example, the photothermal therapy (PTT) exhibits several advantages, such as precise light control on tumors, non-invasive penetration, and low toxicity to normal cells [19, 20]. To combine the nanoparticles with PTT, the photothermal agents (e.g., gold nanorods, carbon nanotubes, polypyrrole (ppy), and graphene oxide) have to be uniformly encapsulated into PNIPAM-based nanoparticles [21–23]. The previous study fabricated the mesoporous silica-coated gold nanorods with a thermo- and pH-responsive PNIPAM shell and further explored in vivo cancer therapy applications [24]. However, these PNIPAM-based nanocomposites require a multi-step synthetic process. In addition, since nanocomposites did not have a targeting moiety against specific cancer cells, it could cause the serious side effects to other organs or normal tissues. In our previous report, we developed a bi-directional controlled release system using the thermo- and pH-sensitive properties of PNIPAM [15]. PNIPAM nanogels were co-polymerized with acrylic acid (AAc) contents for efficient control of LCST. To better enhance cancer targeting and therapeutic efficacy, we developed the cancer targeting combination therapy using chemo- and photothermal effect (Scheme 1). As temperature raises by NIR laser irradiation, the drugs from thermo-responsive PNIPAM nanocomposites were released and photothermal effect was subsequently activated. Due to the conjugated folic acid (FA), PNIPAM-based nanocomposites displayed significantly enhanced therapeutic efficacy toward MDA-MB-231 breast cancer cells.

## Materials and Methods

### Materials

NIPAM, *N,N*′-methylenebisacrylamide (MBA), potassium persulfate (KPS, 99%), N-(3-Dimethylaminopropyl)-*N*′-ethylcarbodiimide hydrochloride (EDC) and *N*-Hydroxysuccinimide (NHS) were purchased from Sigma-Aldrich (St. Louis, MO, USA). Polyvinylpyrrolidone (PVP) and pyrrole (98%) were obtained from Alfa Aesar (Ward Hill, MA, USA). AAc was purchased from Dae Jung Chemicals & Materials. Co. Ltd (Korea). FA-PEG-amine (FA-PEG-NH_2_, MW: 5 kDa) was provided by Nanocs, Inc. (New York, NY, USA). Doxorubicin hydrochloride (Dox) was obtained from Tokyo Chemical Industry. Co. Ltd (Tokyo, Japan). All the chemicals and materials were commercially used without further purification.

### Synthesis of ppy-Decorated PNIPAM-AAc Nanoparticle

The thermo-responsive PNIPAM copolymerized with AAc nanoparticle was synthesized according to previous report [15]. 1.13 g of NIPAM monomer, 0.077 g of MBA and 0.136 g of AAc were dissolved in 100 mL deionized water and added in a 250-mL three-neck round-bottom flask. After 30 min, the reaction temperature was increased to 80 °C and vigorous stirring for 1 h. To induce polymerization, KPS (1.5 mg) was added to the mixture, followed by stirring for 4 h. The mixture was dialyzed against a molecular weight cut off (MWCO) 12–14 kDa dialysis membrane in deionized water for 7 days to remove unreacted monomer, initiator, dispensable ions and PNIPAM-AAc nanoparticle was obtained by freeze-drying for 48 h. To functionalize the photothermal effect, ppy was decorated into PNIPAM-AAc nanoparticles [25]. PVP (50 and 100 mg) and pyrrole monomer (50 and 100 μL) were rapidly added in 10 mg/mL of PNIPAM-AAc solution and were stirred for 12 h at room temperature. Then, KPS (3.4 mg) was inserted in the PNIPAM-AAc solution and was additionally stirred for 14 h. The ppy-decorated PNIPAM-AAc was centrifuged three times with distilled water. The PNIPAM-ppy-5 and PNIPAM-ppy-10 were obtained by freeze-drying for 48 h.

### Synthesis of Cancer Targeted PNIPAM-ppy Nanocomposites

To obtain cancer targeted PNIPAM-ppy nanocomposites (PNIPAM-ppy-FA), 10 mg of PNIPAM-ppy was dissolved in phosphate buffered saline (PBS, 10 mL, pH 5.5) and was ultrasonicated for 5 min. EDC (15 mg, 0.078 mmol) and NHS (15 mg, 0.13 mmol) were added into the PNIPAM-ppy solution. After 1 h, FA-PEG-NH_2_ (5 mg) was added and was additionally stirred for overnight. Unreacted FA-PEG-NH_2_, EDC and NHS in PNIPAM-ppy solution were removed by a dialysis membrane (MWCO 6–8 kDa) and PNIPAM-ppy-FA nanocomposites were lyophilized for 48 h.

### Synthesis of Anticancer Drug-Loaded PNIPAM-ppy-FA Nanocomposites

10 mg of PNIPAM-ppy-FA was dissolved in deionized water and was ultrasonicated for 5 min. 0.5 mg/mL Dox was added in drops to PNIPAM-ppy-FA solution, stirring vigorously at room temperature. Un-loaded Dox was removed by centrifugation (14,000 rpm, 10 min) and was purified with deionized water. Dox-loaded PNIPAM-ppy-FA (Dox@PNIPAM-ppy-FA) was obtained by freeze-drying for 48 h.

### Characterization of NIR and Thermo-Responsive PNIPAM-ppy-FA Nanocomposites

Transmission electron microscopy (TEM, JEOL-2100F, JEOL, Japan) was used to characterize the size, morphology, and distribution of PNIPAM-ppy-FA nanocomposites. For TEM measurement, samples were prepared by placing a drop of the sample solution in deionized water (concentration: 1 g/L) onto a 200-mesh copper grid coated with carbon. The size-distribution and surface charge of PNIPAM-ppy-FA nanocomposites, which were dissolved into deionized water and ultrasonicated for 5 min, were measured by using a Zetasizer Nano Z (Malvern Instruments, UK). The surface modification and chemical bonding of PNIPAM-ppy-FA nanocomposites were confirmed by Fourier transform infrared spectroscopy (FT-IR). FT-IR spectra were recorded on the KBr pellets by using a Nicolet iS50 instrument (Thermo Fisher Scientific, Inc., USA) in the range of 400–4000 cm^−1^ at a resolution of 4 cm^−1^. The optical property and Dox loading capacity in PNIPAM-ppy-FA nanocomposites were observed by UV–Vis spectroscopy (UV 1800, Shimazu, Japan).

### Photothermal Property and Photostability of NIR and Thermo-Responsive PNIPAM-ppy-FA Nanocomposites

The photothermal property of Dox-loaded PNIPAM-ppy-FA nanocomposites (Dox@PNIPAM-ppy-FA) was evaluated by real-time detecting temperature changes. Dox@PNIPAM-ppy-FA was dissolved in aqueous solution with different concentrations of 0.05, 0.1, and 0.2 mg/mL and was irradiated for 20 min using an 808 nm NIR laser (MDL-N-808, CNI Optoelectronics Tech. Co. Ltd., China) at a power density of 2 W/cm^2^. The influence of NIR laser power density was studied by irradiation of NIR laser for 20 min with a power density of 1, 2, and 3 W/cm^2^, respectively. Furthermore, to investigate photothermal stability against NIR laser, Dox@PNIPAM-ppy-FA solution (0.1 mg/mL) was exposed for 15 min and following natural cooling process for five times. During NIR laser irradiation, the temperature of Dox@PNIPAM-ppy-FA solution was measured by a thermo-couple linked to a digital thermometer (DTM-318, Tecpel Co., Taiwan) every 60 s for 15 min.

### NIR and Thermo-Responsive Drug Release of PNIPAM-ppy-FA Nanocomposites

To study the release profiles of Dox by thermo-response, Dox@PNIPAM-ppy-FA solutions (1 mg/mL) were prepared into 5 mL vial and were then stirred at 25, 37 and 50 °C. At a defined release time (0–72 h), the supernatant of each sample was collected by centrifugation and was replaced with an equal volume of fresh medium. The amount of Dox released from PNIPAM-ppy-FA nanocomposites was estimated by measurement of UV–Vis spectroscopy at 480 nm. Additionally, Dox release behavior via NIR laser stimuli-responded Dox@PNIPAM-ppy-FA solution (1 mg/mL) was vigorously stirred at 37 °C and was irradiated for 10 min at pre-determined time points (1, 2, 3, 4, and 5 h). As a control, Dox@PNIPAM-ppy-FA solution without NIR laser irradiation was used. The released Dox was determined by aforementioned method.

### Cytotoxicity Analysis of PNIPAM-ppy-FA Nanocomposites

The cytotoxicity of the PNIPAM-ppy-FA nanocomposites including PNIPAM-ppy-FA and Dox@PNIPAM-ppy-FA was verified by using MTT assay. A549 and MDA-MB-231 cells were seeded in 96-well plates at a density of 1 × 10^4^ cells per a well in 200 µL of RPMI 1640 medium containing 10% FBS and 1% penicillin–streptomycin and were incubated at 37 °C under a humidified atmosphere with 5% CO_2_. After 1 day, 200 µL of PNIPAM-ppy-FA nanocomposites with various concentrations (20–100 µL) was treated to each cell and the plate was incubated for 24 h. The cells were washed with DPBS and the medium was then replaced with fresh medium with MTT agent (0.5 mg/mL). After another incubation for 4 h, the medium was carefully removed and 200 µL of DMSO was added to each well to dissolve the internalized purple formazan crystals. The absorption was measured at 595 nm using an iMark™ microplate reader (Bio-rad, Hercules, CA, USA).

### Cellular Uptake Analysis of PNIPAM-ppy-FA Nanocomposites

To evaluate the targeting ability to specific cancer cells of PNIPAM-ppy-FA nanocomposites, A549 and MDA-MB-231 cells were seeded in a 8-well plate (ibidi, Munich, Germany) at a density of 2 × 10^4^ cells/mL and were incubated for 24 h. The cells were then incubated with PNIPAM-ppy-FA nanocomposites (60 µg/mL) for 6 h. Subsequently, the cells were washed twice with DPBS to remove the remaining nanocomposites and were fixed with 4% paraformaldehyde for 15 min. After treatment of 0.1% Triton-X for 15 min at room temperature, the cells were finally stained by Alexa Fluor 488 phalloidin (1:200, Invitrogen, USA) for 1 day at 4 °C and 4,6-diamidino-2-phenylindole (DAPI, Thermo Fisher Scientific, USA) for 10 min, respectively. The cellular uptake images were observed by using a confocal laser scanning microscopy (CLSM, LSM 710, Carl Zeiss, Germany).

### Enhanced Anticancer Effects of PNIPAM-ppy-FA Nanocomposites via NIR Laser Irradiation

The enhanced therapeutic efficacy using NIR and thermo-responsive PNIPA-ppy-FA on breast cancer cells were investigated by MTT assay. MDA-MB-231 (1 × 10^4^ cells/mL) were seeded onto 96-well plates and were incubated for 24 h. The cells were then washed with DPBS and PNIPAM-ppy-FA and Dox@PNIPAM-ppy-FA nanocomposites at various concentrations (20, 40, 60, 80 and 100 μg/mL) were added into each well in a concentration-dependent manner. After incubation for overnight, the medium was removed, followed by the addition of fresh medium. For the NIR laser irradiation groups, the cells were treated with 5 W/cm^2^ for 5 min. Then, MDA-MB-231 were washed with DPBS and the fresh medium including MTT solution was added. After 4 h, the medium was carefully removed and 200 μL of DMSO was added into the each well. Finally, the absorbance at 595 nm was measured by a iMark™ microplate reader to determine the cell viability. As an another method for observation of cancer therapeutic effects, the live and dead assay was carried out. PNIPAM-ppy-FA and Dox@PNIPAM-ppy-FA (60 μg/mL) were treated into MDA-MB-231. After 24 h, MDA-MB-231 were exposed with or without NIR laser irradiation (5 W/cm^2^, 5 min) and were stained with calcein AM and ethidium homodimer-1. After 30 min, the cells were washed several times with DPBS. The live/dead images were obtained by using inverted fluorescence microscopy (Olympus Ix73, Japan).

## Results and Discussion

### Synthesis and Characterization of NIR and Thermos-Responsive Cancer Targeted PNIPAM-ppy Nanocomposites

**Fig. 1:**
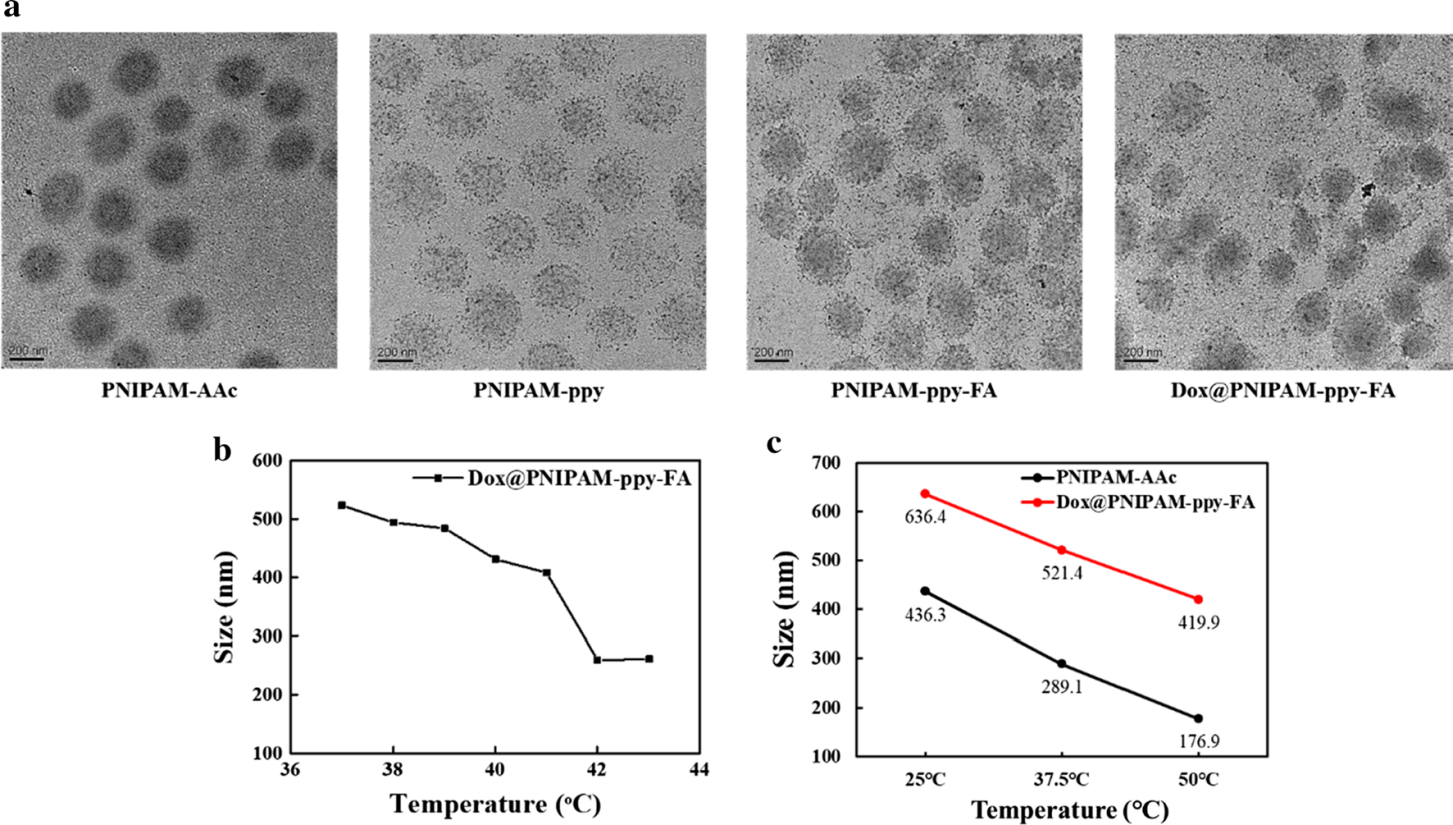
**a** TEM images of PNIPAM-AAc, PNIPAM-ppy, PNIPAM-ppy-FA, and Dox@PNIPAM-ppy-FA nanocomposites. **b** LCST analysis of Dox@PNIPAM-ppy-FA by DLS. **c** Average diameters of PNIPAM-AAc and Dox@PNIPAM-ppy-FA depending on different temperature conditions (at 25 °C, 37.5 °C, and 50 °C)

For the generation of thermo-responsive PNIPAM-based nanocomposites, PNIPAM-AAc nanoparticles were fabricated by a radical polymerization method [15]. To control LCST via NIR laser irradiation, ppy was covered by polymerization reaction in as-prepared PNIAPM-AAc. The cancer targeted PNIPAM-ppy-FA was synthesized by chemically conjugating FA-PEG-NH_2_ to the carboxyl group in PNIPAM-AAc. According to TEM images in Fig. 1a, the morphology, size, and dispersity of PNIPAM-AAc, PNIPAM-ppy and PNIPAM-ppy-FA were observed. As-prepared PNIPAM-AAc exhibited the homogeneous form with an average diameter of 274.32 ± 11.62 nm. After decoration of the ppy, PNIPAM-ppy and PNIPAM-ppy-FA showed the similar sizes with 275.99 ± 11.41 and 285.77 ± 17.92 nm. The size of Dox-loaded PNIPAM-ppy-FA increased slightly from 285.77 ± 17.92 to 290.73 ± 12.28 nm, indicating that the anticancer drugs were loaded in PNIPAM-ppy-FA nanocomposites [26]. Interestingly, as compared with PNIPAM-AAc, the presence of the ppy within PNIPAM-ppy-FA nanocomposites was clearly exhibited by small black spots. In addition, due to the PEG chain, PNIPAM-ppy-FA nanocomposites were well dispersed in aqueous solution without aggregation [27]. The average hydrodynamic diameters of PNIPAM-AAc, PNIPAM-ppy, PNIPAM-ppy-FA, and Dox@PNIPAM-ppy-FA were measured by DLS analysis (Additional file 1: Fig. S1). The diameters of PNIPAM-AAc, PNIPAM-ppy, PNIPAM-ppy-FA, and Dox@PNIPAM-ppy-FA were 432, 450.7, 468.6, and 486.7 nm, respectively. Due to different analytical methods, the diameter of each nanocomposite was larger than that of TEM, but the particle size and distribution of PNIPAM-ppy-FA nanocomposites exhibited a similar trend.

To investigate the influence of polymerized ppy into PNIPAM-AAc, the LCST values of PNIPAM-AAc and Dox@PNIPAM-ppy-FA were measured by different temperatures (Fig. 1b). Compared to our previous work [15], the LCST of Dox@PNIPAM-ppy-FA was slightly decreased than that of PNIPAM-AAc (42 °C). This difference could be caused by the reduced carboxyl groups in PNIPAM-AAc owing to polymerized ppy and conjugated cancer targeting ligand [28]. Subsequently, we monitored the change of particle size depending on the temperature in Fig. 1c. For this, the diameters of PNIPAM-AAc and Dox@PNIPAM-ppy-FA were measured in the temperature range from 25 to 50 °C. As temperature was increased, PNIPAM-AAc was decreased from 436 to 177 nm. Moreover, the size of Dox@PNIPAM-ppy-FA was greatly reduced from 630 to 420 nm, indicating that ppy-decorated in PNIPAM nanocomposites did not highly affect for applications of controlled and thermo-responsive DDS [27].

**Fig. 2:**
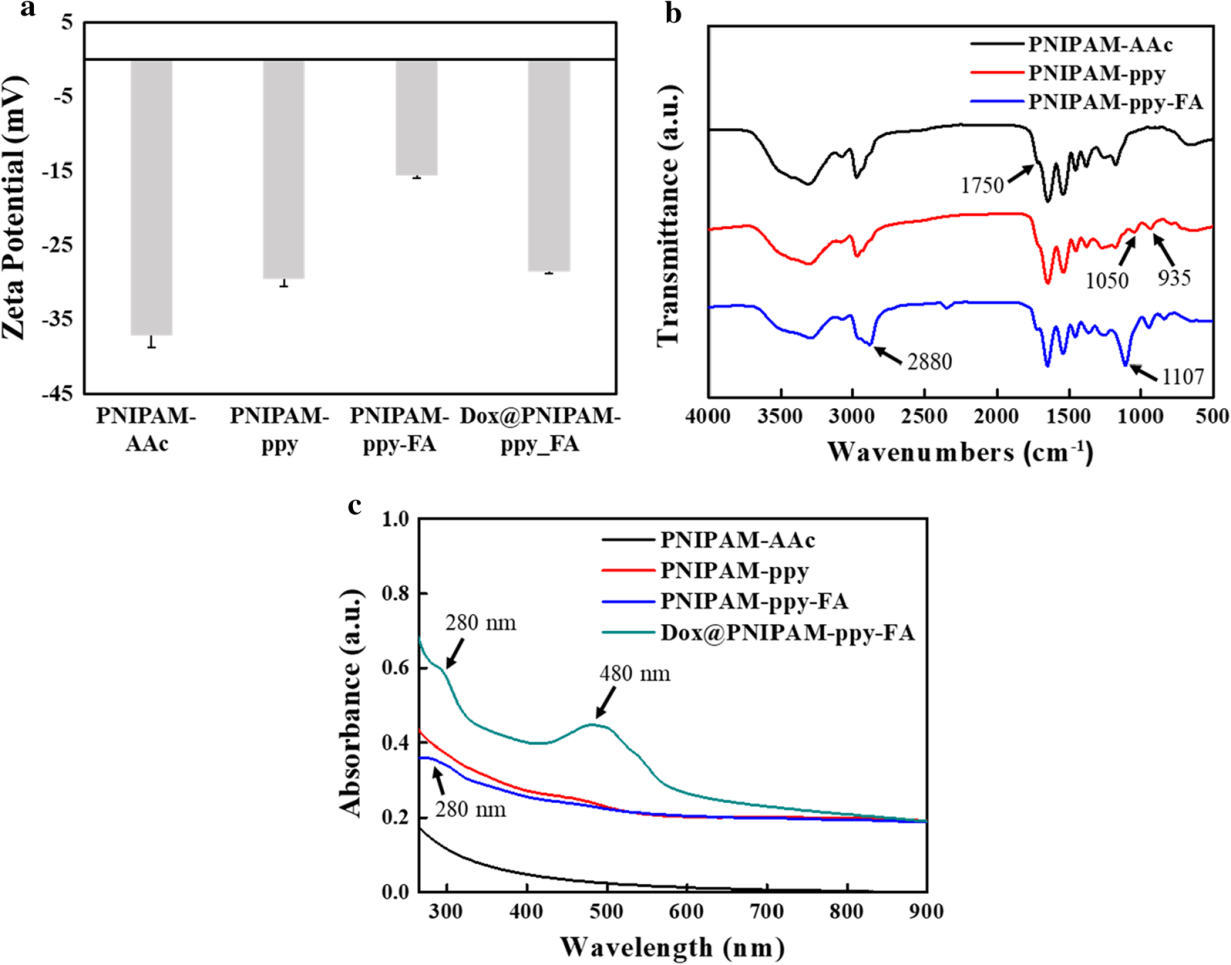
**a** Zeta potential analysis of PNIPAM-AAc, PNIPAM-ppy, PNIPAM-ppy-FA, and Dox@PNIPAM-ppy-FA. **b** FT-IR spectra of PNIPAM-AAc, PNIPAM-ppy, and PNIPAM-ppy-FA. **c** UV–Vis spectra of PNIPAM-AAc, PNIPAM-ppy, PNIPAM-ppy-FA, and Dox@PNIPAM-ppy-FA

The zeta potential values of PNIPAM-AAc, PNIPAM-ppy, PNIPAM-ppy-FA, and Dox@PNIPAM-ppy-FA displayed the surface change of nanocomposites before and after modification (Fig. 2a). The zeta potential of PNIPAM-AAc was − 37.1 ± 1.61 mV due to the carboxyl groups in AAc [29]. The values of PNIPAM-ppy and PNIPAM-ppy-FA increased to − 29.6 ± 0.96 and − 15.6 ± 0.26 mV, indicating that positively charged polypyrrole and FA-PEG-NH_2_ were successfully introduced in PNIPAM-ppy-FA nanocomposites [27]. Due to the negatively charged Dox, zeta potential of Dox@PNIPAM-ppy-FA changed to more negative charges (− 28.6 ± 0.23 mV). As shown in Fig. 2b, the successful synthesis of PNIPAM-AAc, PNIPAM-ppy, and PNIPAM-ppy-FA was confirmed by FT-IR spectroscopy. The spectrum of PNIPAM-AAc showed the stretching vibration peaks of C-N and CH_2_ between 1100 and 1200 cm^−1^ and the peaks of C=O, N–H and COOH were observed at 1545, 1645 and 1750 cm^−1^, which belonged to PNIPAM-AAc [15]. The polypyrrole in PNIPAM-ppy and PNIPAM-ppy-FA demonstrated the additional peaks at 935 and 1050 cm^−1^ were observed [30]. In spectra of PNIPAM-ppy-FA, the new vibration peaks appeared at 1107 and 2880 cm^−1^, which attributed to C–O–C of PEG chain. This result indicated the successful chemical conjugation of FA-PEG-NH_2_ [31]. However, the targeting ligand of FA was not detected by FT-IR, because its structure was similar to PEG. To confirm the presence of FA in PNIPAM nanocomposites, PNIPAM-AAc, PNIPAM-ppy, and PNIPAM-ppy-FA were performed by UV–Vis spectroscopy (Fig. 2c). While the spectra of PNIPAM-AAc and PNIPAM-ppy (without FA) did not show any absorption peak, PNIPAM-ppy-FA showed the additional peak at 280 nm, which was distinctive in FA [32]. This result supported that FA molecules were grafted on PNIPAM-based nanocomposites.

### Photothermal Property of PNIPAM-ppy-FA Nanocomposites via NIR Laser Irradiation

**Fig. 3:**
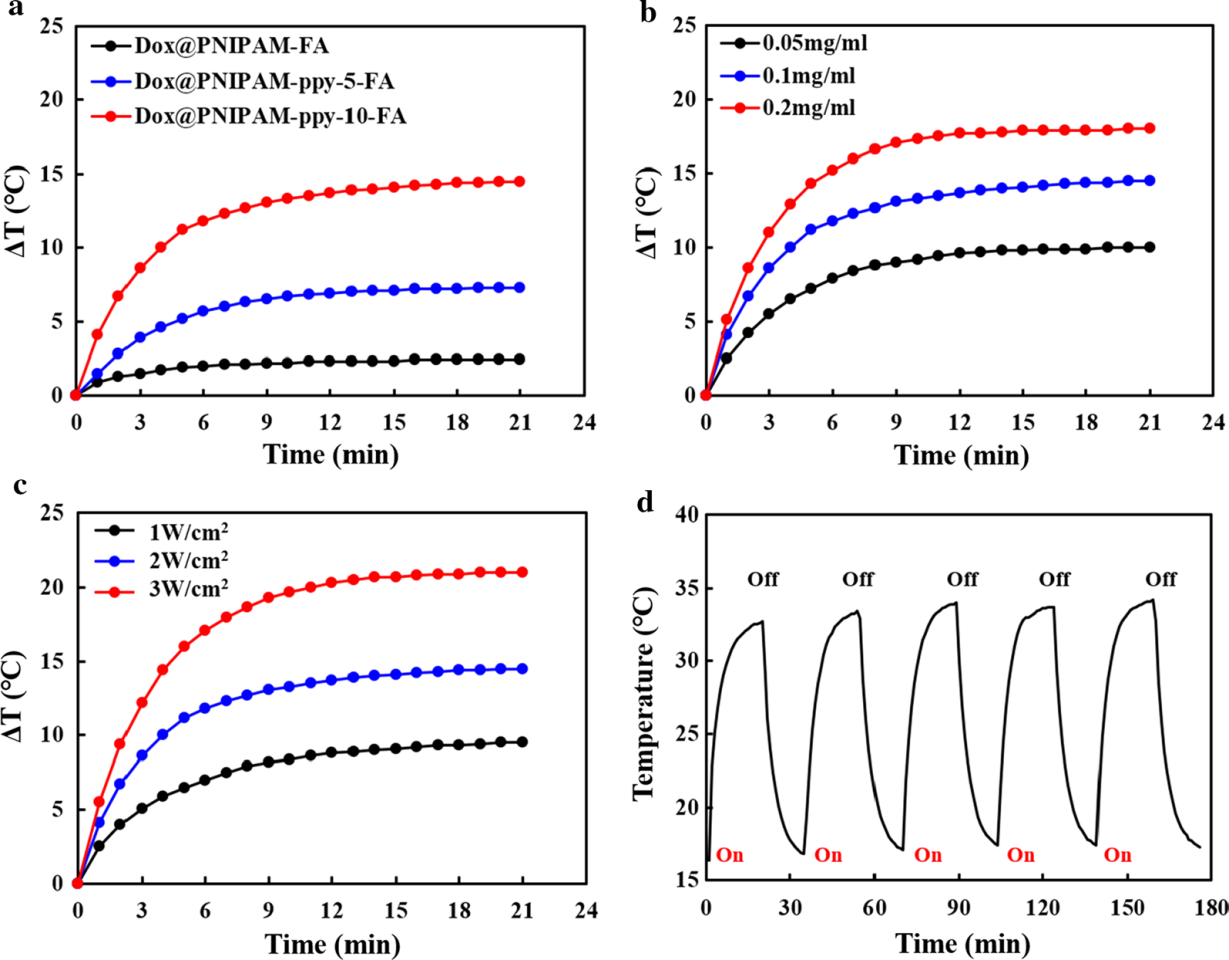
Photothermal performance of PNIPAM-ppy-FA nanocomposites. **a** Temperature analysis of Dox@PNIPAM-ppy-5-FA and Dox@PNIPAM-ppy-10-FA under 808 nm NIR laser irradiation (2 W/cm^2^). **b** Temperature analysis of different concentration of Dox@PNIPAM-ppy-FA under 808 nm NIR irradiation (2 W/cm^2^). **c** Photothermal effect of Dox@PNIPAM-ppy-FA with various power densities (1, 2, and 3 W/cm^2^). (D) Temperature curve of Dox@PNIPAM-ppy-FA over five on/off cycles using NIR laser irradiation (2 W/cm^2^)

To use PNIPAM-based nanocomposites for photothermal therapy, the optical property of PNIPAM-ppy-FA nanocomposites were examined by UV–Vis spectroscopy. As shown in Fig. 2c, PNIPAM-AAc did not show any absorbance in NIR region (λ = 700–1000 nm). However, the spectra of PNIPAM-ppy, PNIPAM-ppy-FA, and Dox@PNIPAM-ppy-FA obviously showed the strong absorbance in the same range due to polymerized ppy nanoparticles. These absorbance results demonstrated that PNIPAM-ppy-FA nanocomposites could convert NIR light into heat [26]. Next, we evaluated photothermal property of PNIPAM-ppy-FA nanocomposites by NIR laser irradiation (Fig. 3). To optimize the photothermal effect, PNIPAM-FA (without pyrrole), PNIPAM-ppy-5-FA (ppy 50 μL), and PNIPAM-ppy-10-FA (ppy 100 μL) were prepared at same concentration (0.1 mg/mL) and then were exposed to 808 nm NIR laser irradiation at a density of 2 W/cm^2^ for 20 min (Fig. 3a). The temperature of PNIPAM-ppy-10-FA increased to 14.5 °C, which was 2 times higher than that of PNIPAM-ppy-5-FA. As a control, PNIPAM-FA without ppy was irradiated, but the temperature was negligibly increased (2.4 °C). Therefore, we chose PNIPAM-ppy-10-FA to investigate additional photothermal experiments. To observe the temperature changes by concentrations, NIR laser was irradiated to PNIPAM-ppy-FA at different concentrations (0.05, 0.1, and 0.2 mg/mL). In Fig. 3b, depending on the concentration, the temperature increased to 10, 14.5, and 18 °C, respectively. Furthermore, PNIPAM-ppy-FA solution (0.1 mg/mL) was irradiated with various laser power densities (1–3 W/cm^2^) in Fig. 3c. As expected, the temperature rise of nanocomposite was highly dependent on the laser power. Subsequently, we observed the photothermal stability of PNIPAM-ppy-FA nanocomposites in Fig. 3d. Although NIR laser irradiation was repetitively performed at least 5 times, the temperature was steadily increased to 33 °C, showing that the PNIPAM-ppy-FA nanocomposite was a suitable nanocarrier as a photothermal therapy.

### NIR and Thermal Responsive Drug Release from PNIPAM-ppy-FA Nanocomposites

**Fig. 4:**
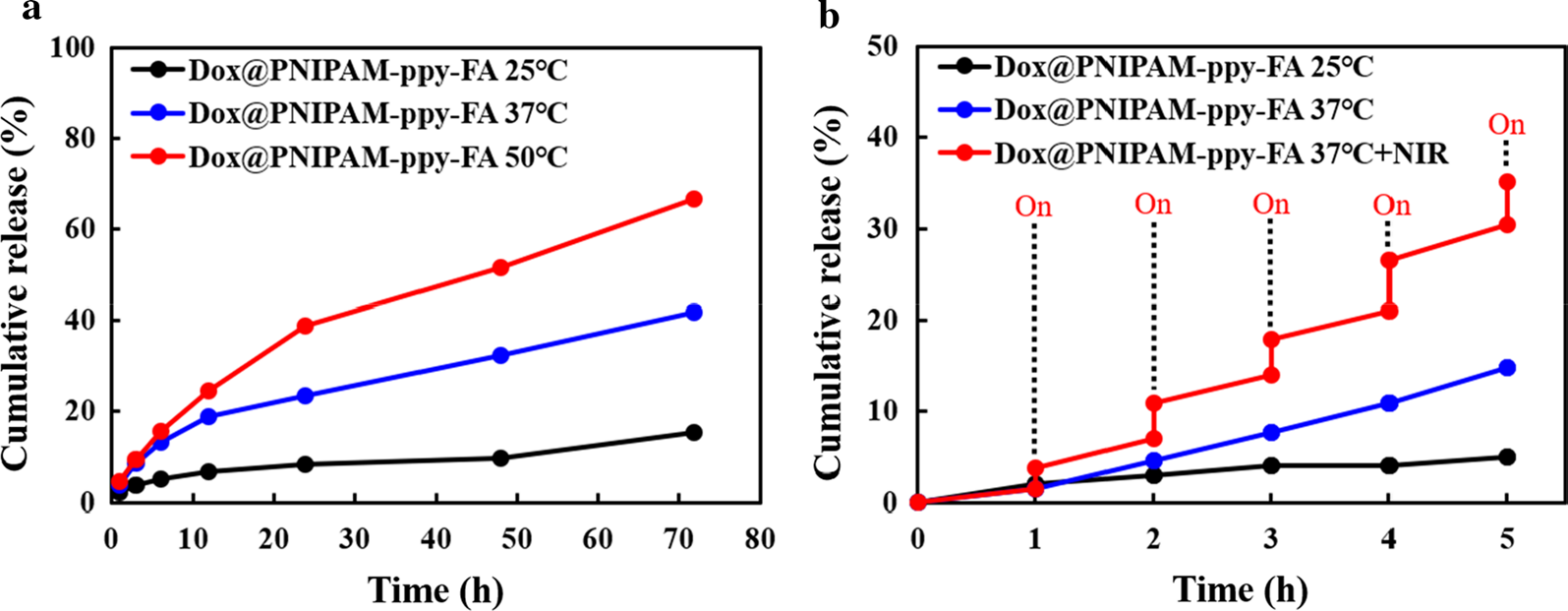
**a** Cumulative Dox release profiles of Dox@PNIPAM-ppy-FA via thermo-response and **b** 808 nm NIR laser irradiation

To evaluate the drug release profiles via NIR and thermo-response, we analyzed the encapsulation and release properties of Dox@PNIPAM-ppy-FA nanocomposites. Initially, we measured UV–Vis absorption spectra of the drug-loaded PNIPAM-ppy-FA and a strong absorption peak was observed near 480 nm (Fig. 2c). This result indicated that Dox successfully loaded into PNIPAM-ppy-FA nanocomposites. The Dox loading efficiency of PNIPAM-ppy-FA nanocomposites was calculated to 15% using Dox calibration curve (data not shown). In Fig. 4a, Dox release profile from PNIPAM-ppy-FA was studied at different temperatures. We observed Dox release behavior for 72 h at 25 °C, 37 °C, and 50 °C and the cumulative release of Dox from PNIPAM-ppy-FA were 15%, 42%, and 67%, respectively. Since LCST of PNIPAM-ppy-FA decreased slightly to 42 °C, the cumulative release at body temperature was found to be relative higher than room temperature. A large amount of Dox was released, however, the released Dox did not affect the therapeutic efficiency, because Dox@PNIPAM-ppy-FA nanocomposites could be internalized in only folate-receptor positive cells, as previously described [26, 32]. At above LCST (50 °C), the amount of Dox from PNIAPM-ppy-FA nanocomposites was released four times higher than room temperature, suggesting that PNIPAM-ppy-FA nanocomposites could be used in controlled drug release system through thermal response. In addition, we investigated the NIR light triggered release behavior of PNIPAM-ppy-FA nanocomposites in Fig. 4b. Dox@PNIAPM-ppy-FA solution (1 mg/mL) was exposed to NIR laser (3 W/cm^2^) irradiation for 10 min and this operation was repeated every 1 h. During 6 h, the total amount of Dox via NIR laser irradiation reached 35%, whereas the Dox release profile without NIR laser irradiation showed about 5% and 15% (25 °C and 37 °C). This result showed that NIR laser irradiation resulted in a higher temperature than LCST of nanocomposites and induced Dox release from the PNIPAM-ppy-FA nanocomposites.

### Cytotoxicity and Cellular Uptake Analysis of PNIPAM-ppy-FA Nanocomposites

According to literature, the cytotoxicity of PNIPAM-ppy-FA nanocomposites was investigated toward lung and breast cancer cells (Additional file 1: Fig. S2) [33, 34]. Regardless of sample concentrations, after incubation with PNIPAM-ppy-FA for 24 h, both cell viabilities were observed (> 85%). Therefore, our PNIPAM-ppy-FA nanocomposites did not show cytotoxicity as a nanocarrier for drug delivery applications [35, 36]. To investigate whether PNIPAM-ppy-FA could selectively deliver to cancer cells, we examined the fluorescent signals of intracellular PNIPAM-ppy-FA nanocomposites using confocal laser scanning microscopy (Additional file 1: Fig. S3). The cellular uptake behaviors of the PNIPAM-ppy-FA and Dox@PNIPAM-ppy-FA were evaluated in folate-receptor negative (A549) and positive (MDA-MB-231) cell lines [37]. After incubating of MDA-MB-231 breast cancer cells with Dox@PNIPAM-ppy-FA, strong red fluorescence was observed, showing that Dox@PNIAPM-ppy-FA were internalized into lysosomes of MDA-MB-231 cells. In constant, when PNIPAM-ppy-FA and Dox@PNIPAM-ppy-FA were treated with A549 cells, Dox signals were not observed. These confocal images demonstrated that PNIAPM-ppy-FA nanocomposites could be selectively internalized into folate-receptor overexpressed cancer cells via receptor-mediated endocytosis pathway as previously described [38, 39].

### Chemo-Photothermal Anticancer Effects of NIR and Thermo-Responsive PNIPAM-ppy-FA Nanocomposites

**Fig. 5:**
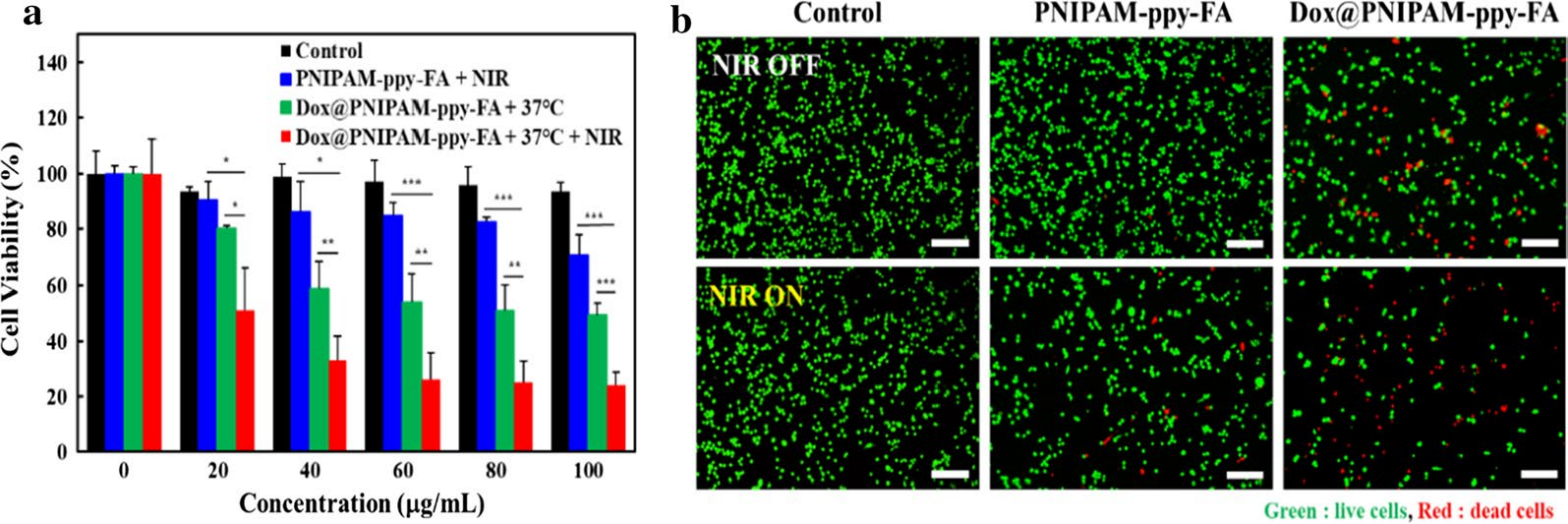
**a** Quantitative analysis of viability of MDA-MB-231 breast cancer cells treated with PNIPAM-ppy-FA and Dox@PNIPAM-ppy-FA with different concentrations and with or without 808 nm NIR laser irradiation. **b** Fluorescence images of live/dead assay in MDA-MB-231 cells after treatment of PNIPAM-ppy-FA and Dox@PNIPAM-ppy-FA nanocomposites (60 μg/mL) with or without NIR laser irradiation. The live and dead cells are stained with Calcein AM (green) and Ethidium homodimer-1 (red). Scale bars are 200 μm

To confirm in vitro combination therapeutic efficacy of PNIPAM-ppy-FA nanocomposites, the PNIPAM-ppy-FA nanocomposites were evaluated on MDA-MB-231 cells with or without NIR laser irradiation and the cell viabilities were evaluated by MTT assay in Fig. 5a. For photothermal therapy, the MDA-MB-231 breast cancer cells with PNIPAM-ppy-FA were incubated for 12 h and were subsequently exposed to NIR laser (5 W/cm^2^, 5 min). The cell viability of MDA-MB-231 breast cancer cells decreased to 70–90% depending on the concentrations of PNIPAM-ppy-FA. To confirm the only chemotherapeutic effect, Dox-loaded PNIPAM-ppy-FA nanocomposites were treated. We observed that the viabilities diminished to 50% due to Dox release from nanocomposites, corresponding to release profile in Fig. 4. Interestingly, after NIR laser irradiation in the MDA-MB-231 cells with Dox@PNIPAM-ppy-FA nanocomposites, the cell viability dramatically decreased up to 24%, which were higher than those of control groups (PNIPAM-ppy-FA with NIR laser irradiation, Dox@PNIPAM-ppy-FA without NIR laser irradiation). This result indicated an optimal synergy effect of NIR laser irradiation-mediated chemo-photothermal combination therapy. Moreover, to directly observe the therapeutic efficiency in cancer cells, the live/dead assay was performed in Fig. 5b. As a control group, we observed the viability of only MDA-MB-231 cells with or without NIR laser irradiation (5 W/cm^2^, 5 min) and most cells showed green fluorescence (live cells). It demonstrated that NIR laser irradiation did not evidently affect the cell viability. In addition, the MDA-MB-231 cells with PNIPAM-ppy-FA under NIR laser irradiation showed a few red fluorescence (dead cells), indicating photothermal therapeutic effect caused from PNIPAM-ppy-FA nanocomposites. Despite no NIR laser irradiation in MDA-MB-231 cells with Dox@PNIPAM-ppy-FA, a few sporadic red fluorescence was observed, indicating chemotherapy. Furthermore, when MDA-MB-231 cells were treated with nanocomposites and NIR laser irradiation, the most cancer cells exhibited a few red fluorescence [39, 40]. These results supported the synergistic therapeutic effects of chemo-photothermal therapy via NIR laser irradiation and thermo-responsive Dox@PNIPAM-ppy-FA nanocomposites.

## Conclusion

We have successfully constructed a cancer targeted NIR and thermo-responsive PNIPAM-ppy-FA nanocomposites for chemo-photothermal combination therapy. To induce the controlled release and photothermal effect via 808 nm NIR laser irradiation, ppy nanoparticles were uniformly decorated on PNIPAM-AAc by polymerization method. Dox-loaded PNIPAM-ppy-FA showed significant photothermal effect and photostability. Furthermore, Dox@PNIPAM-ppy-FA showed favorable properties of thermo-sensitive transition at different conditions and the drug release from PNIPAM-ppy-FA nanocomposites could be controlled by NIR light and thermo-response. In vitro studies verified that PNIPAM-ppy-FA nanocomposites showed excellent biocompatibility and enhanced therapeutic efficacy toward breast cancer cells. This enhanced anticancer efficacy via nanocomposites was contributed to the following reasons: (1) specific cellular uptake of nanocomposites in folate-receptor mediated endocytosis, (2) accumulated Dox release from PNIPAM-ppy-FA via NIR and thermo-response, and (3) synergistic therapeutic effect by chemo-photothermal combination. Therefore, our PNIPAM-ppy-FA nanocomposite could be potentially used as a multi-functional nanocarrier for synergistic therapeutic approaches toward different types of cancers with reduced side effect.

## Supplementary information

**Additional file 1:** **Fig. S1.** Size distribution of PNIPAM-AAc, PNIPAM-ppy, PNIPAM-ppy-FA, and Dox@PNIPAM-ppy-FA nanocomposites in an aqueous solution at room temperature. **Fig. S2.** (A) Cytotoxicity analysis of PNIPAM-ppy-FA nanocomposites against A549 lung cancer and MDA-MB-231 breast cancer cells. (B) Cytotoxicity analysis of PNIPAM-ppy-FA and Dox@PNIPAM-ppy-FA in A549 lung cancer cells without NIR laser irradiation. **Fig. S3.** Intracellular uptake images of folate receptor targeting of PNIPAM-ppy-FA and Dox@PNIPAM-ppy-FA to (A, B) A549 lung cancer cells and (C) MDA-MB-231 breast cancer cells treated with Dox@PNIPAM-ppy-FA. Green, red, and blue represent phalloidin-stained cytoplasm, Dox fluorescence and DAPI-stained cell nuclei, respectively. Scale bars are 50 μm.

## Data Availability

The data and the analysis in the current work are available from the corresponding authors on reasonable request.

## Abbreviations

PTT: Photothermal therapy
NIR: Near-infrared
FA: Folic acid
FA-PEG-NH_2_: FA-PEG amine
LCST: Lower critical solution temperature
PNIPAM: Poly(*N*-isopropylacrylamide)
ppy: Polypyrrole
TEM: Transmission microscopy
UV–Vis: Ultraviolet–visible spectroscopy
