# Supplementary material for: Near-Infrared Light-Triggered Thermo-responsive Poly(N-Isopropylacrylamide)-Pyrrole Nanocomposites for Chemo-photothermal Cancer Therapy
Source: Nanoscale Res Lett. 2020 Nov 12;15:214. doi: 10.1186/s11671-020-03444-4 (PMC7661614; doi:10.1186/s11671-020-03444-4)
Supplement: Supplementary file 1 — Additional file 1: Fig. S1. Size distribution of PNIPAM-AAc, PNIPAM-ppy, PNIPAM-ppy-FA, and Dox@PNIPAM-ppy-FA nanocomposites in an aqueous solution at room temperature. Fig. S2. (A) Cytotoxicity analysis of PNIPAM-ppy-FA nanocomposites against A549 lung cancer and MDA-MB-231 breast cancer cells. (B) Cytotoxicity analysis of PNIPAM-ppy-FA and Dox@PNIPAM-ppy-FA in A549 lung cancer cells without NIR laser irradiation. Fig. S3. Intracellular uptake images of folate receptor targeting of PNIPAM-ppy-FA and Dox@PNIPAM-ppy-FA to (A, B) A549 lung cancer cells and (C) MDA-MB-231 breast cancer cells treated with Dox@PNIPAM-ppy-FA. Green, red, and blue represent phalloidin-stained cytoplasm, Dox fluorescence and DAPI-stained cell nuclei, respectively. Scale bars are 50 μm. [file 11671_2020_3444_MOESM1_ESM.docx]

**Supporting information**

**
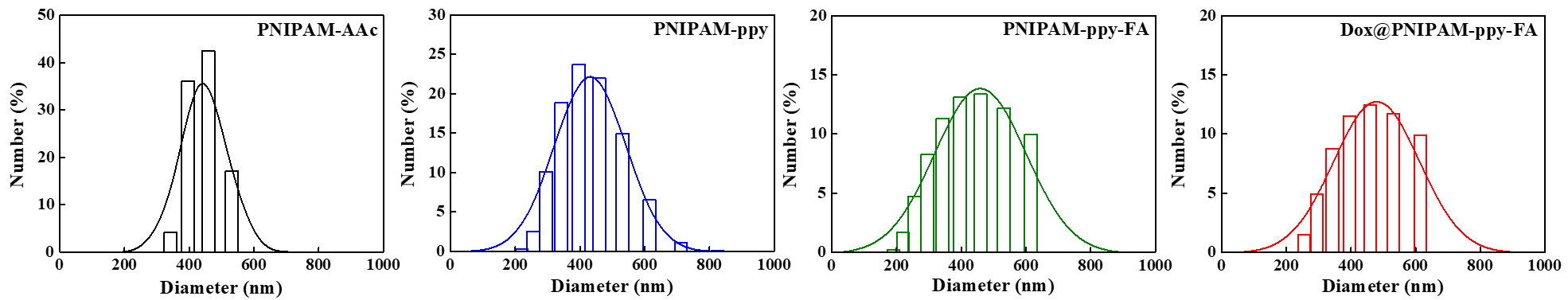
**

**Figure S1.** Size distribution of PNIPAM-AAc, PNIPAM-ppy, PNIPAM-ppy-FA, and Dox@PNIPAM-ppy-FA nanocomposites in an aqueous solution at room temperature.

**
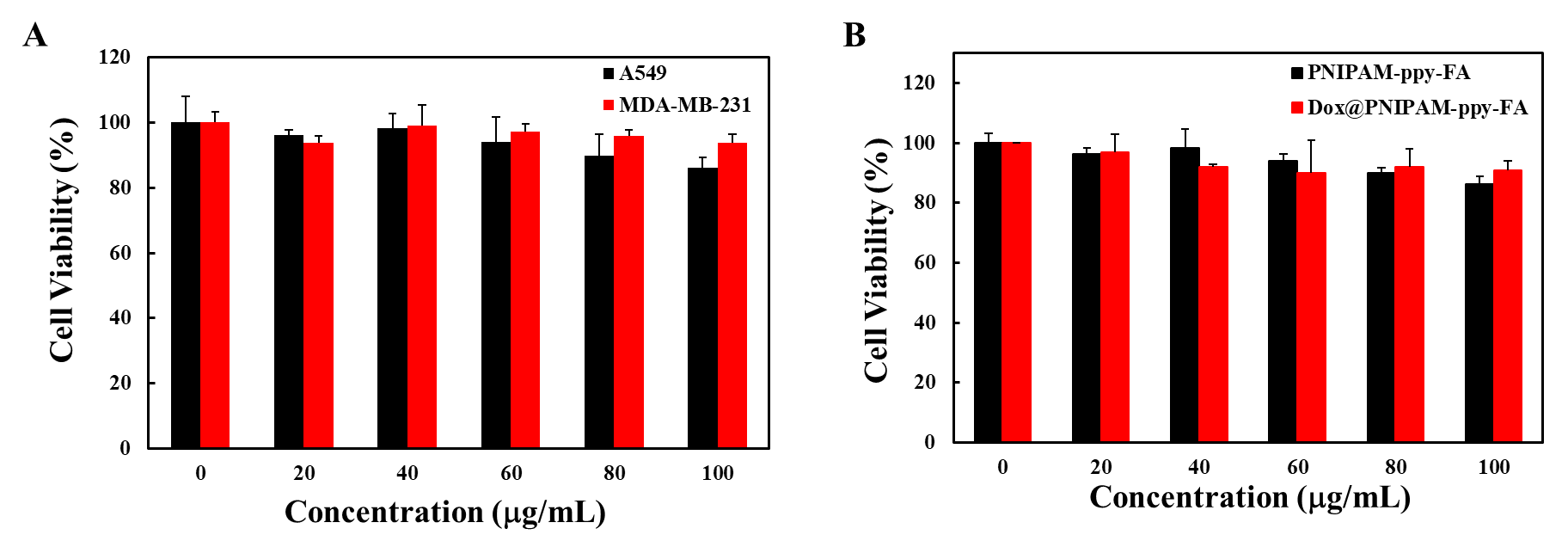
**

**Figure S2.** (A) Cytotoxicity analysis of PNIPAM-ppy-FA nanocomposites against A549 lung cancer and MDA-MB-231 breast cancer cells. (B) Cytotoxicity analysis of PNIPAM-ppy-FA and Dox@PNIPAM-ppy-FA in A549 lung cancer cells without NIR laser irradiation.


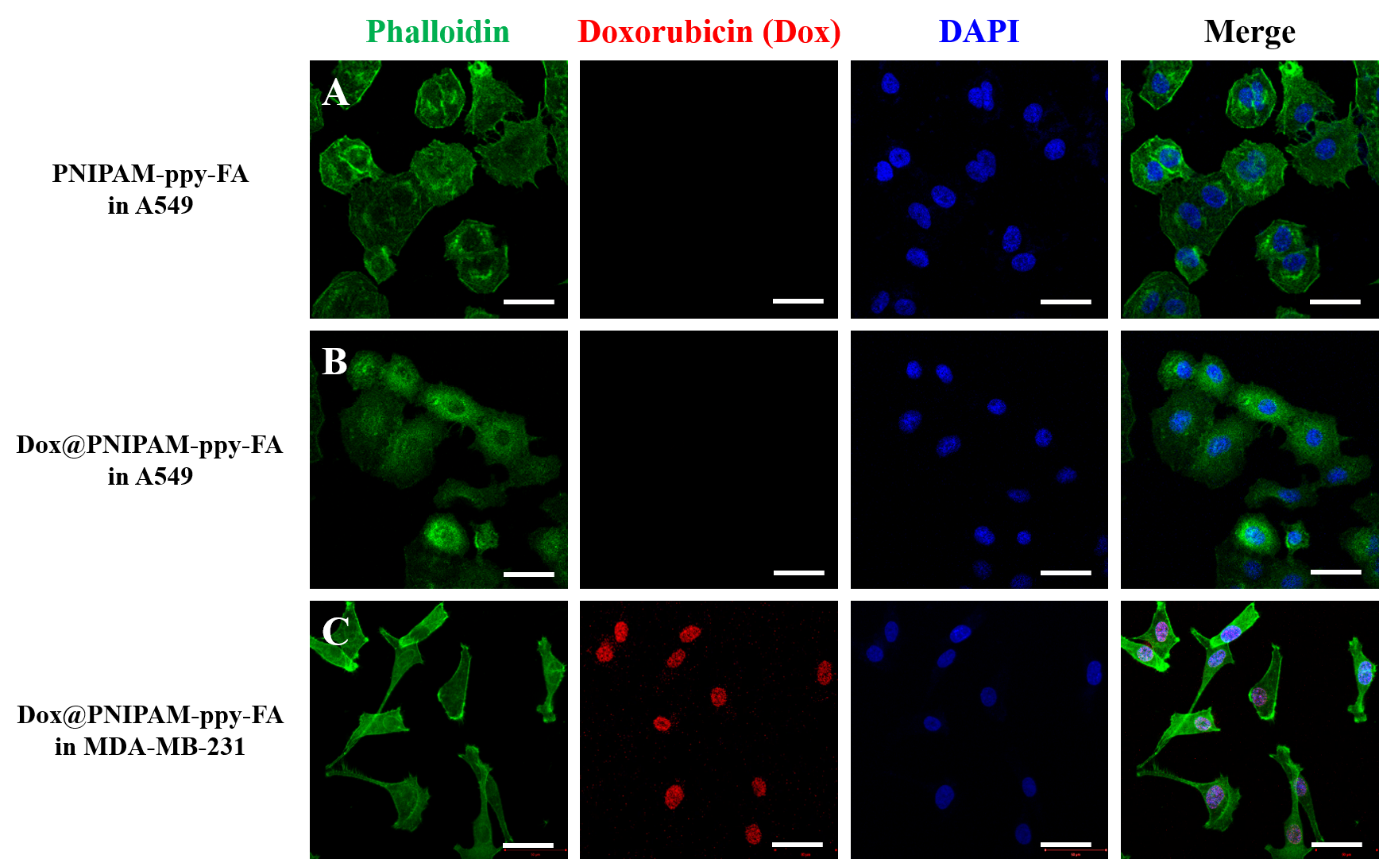
**Figure S3.** Intracellular uptake images of folate receptor targeting of PNIPAM-ppy-FA and Dox@PNIPAM-ppy-FA to (A, B) A549 lung cancer cells and (C) MDA-MB-231 breast cancer cells treated with Dox@PNIPAM-ppy-FA. Green, red, and blue represent phalloidin-stained cytoplasm, Dox fluorescence and DAPI-stained cell nuclei, respectively. Scale bars are 50 μm.
